# The Field of Science

**Published:** 1894-01-20

**Authors:** 


### The Field of Science.

An Anti-Rabic Institute was opened at Marseilles in December; it is located in the Chateau de Pharo, and is under the direction of the Marseilles School of Medicine.

We mentioned in these pages not long since, that Algeria, of all foreign countries, had supplied the greatest number of subjects seeking relief at the Pasteur Institute in Paris. We now see official announcement of the fact that a branch establishment of that institute will be opened at Algiers next year.

A curious fact has been noted at the London County Asylum, Banstead. It appears that the percentage of recoveries of the inmates has gone up since the withdrawal of beer from the dietary. Perhaps the evidence thus furnished is not quite sufficient to be entirely conclusive, but at this particular institution we hear that since the suspension of the use of intoxicating liquors the recoveries of patients reached 46·97 per cent., nearly one half of the total number of inmates having recovered during the period thus stated.

Dr. A. C. Abbott, working in the Laboratory of Hygiene at the University of Philadelphia, carried out some peculiarly interesting and important experiments on milk cows, with the view of determining, among other things, whether or not the *bacillus diphtheriæ* was actually present in the milk of cows suffering from diphtheria. Certain cows were inoculated with cultures of the bacillus diphtheriæ, and in due course took diphtheria; or, at any rate, an acute febrile disease resembling it, and either died or were killed. What was the condition of the milk of these animals when actually suffering from diphtheria? By way of ascertaining how far the milk varied from the normal, Dr. Abbott took the precaution of examining each cow's milk for nine days before the date of inoculation. After the inoculations in each case the milk was examined every day also for nine days. The result, so far as the presence of *bacillus diphtheriæ* in the milk was concerned, was practically *nil*.

"The studies," says Dr. Abbott, "upon the milk which, as stated covered nine days succeeding the inoculation, were negative, in so far as finding the bacilli that had been injected into the tissues of the animal were concerned. As in the studies made before inoculation, there appeared occasionally bacilli which in morphology alone were a little suggestive of the *B. diphtheriæ*, but which could easily be differentiated from it by culture tests." This is so far reassuring. But one cannot help thinking that the research would have been greatly increased in practical value if some of the milk, notwithstanding the absence of *bacillus diphtheriæ*, had been injected into other animals, such as dogs, cats, or rabbits, and also given to them as food. For although no actual bacilli were found, it is quite possible that the milk might have undergone such chemical changes in the udders of the infected cows as to render it poisonous in a greater or less degree to animal life.

Yale College has undertaken to organise a series of experiments with a view to determining the relations of the nerves to the muscles of the human body. These tests were projected in substantiation of a new theory that physical strength depends less upon the size of the muscle than upon the strength of the nerve.

Let us hope the Manchester Ship Canal will not be called upon to fulfil the twofold mission which for some time past its less ambitious predecessor canals have done. Canals which pass through towns and other inhabited quarters have ever been the recipients for the refuse of such houses as are located on their banks. Anything and everything is thrown in on the supposition, we may charitably presume, on the part of the throwers that the "stream" would bear away whatsoever impurities are thus consigned to its waters. But of all canals, has any been more grossly abused than a certain one in the metropolis? The finger of scorn has long been pointed at the Regent's Canal, and with reason. Originally designed for navigable purposes, the engineers did not include in their plans for this any special thought of public sewer purposes which it would soon be called upon to fulfil.

But the condition of this canal has developed into a positive danger, menacing public health. The Zoological Gardens drain into it; at the Paddington basin alone an average of 500 sleep on its boats each night, which means, in other words, that the sewerage of a large village is emptied into it, and deposited in its waters. The stagnant waterway is little else but a sewer in such parts as we have alluded to. The difficulties of putting a stop to this state of things can well be imagined when we remember that the Regent's Canal passes through no less than fifteen metropolitan districts, and it is readily to be surmised a general consensus of opinion of all concerned is not easily arrived at. The matter, however, has at length been carefully looked into, and a conference has been called wherein representatives and medical officers from all the sanitary authorities concerned will check the growth of this serious public evil.

An account of the following tragic circumstance, which occurred quite recently, comes to us from Russia. Cholera, it appears, had broken out in the neighbourhood of a certain village called Tomsk, the inhabitants of which place, anxious to insure immunity from the ravages of this disease in their midst, set up for themselves a code of precautionary measures. Amongst other rules in this direction was one forbidding the entrance of any stranger from outside to any part of their little hamlet. One "Feast Day" the inhabitants of Tomsk were surprised by the arrival of a strange woman on the scene of their festivities. The benighted peasants gave chase to the intruder, and, believing that in her person she represented "Cholera" itself, they sacrificed the woman to their superstitions. After her death a solemn mass was offered in thanksgiving to God for having delivered them from the terrible scourge which had come amongst them "disguised" in the form of a female. The Government authorities have viewed the matter in another light, and two of the ignorant perpetrators of this crime have had a severe sentence passed upon them.

